# Finding combinatorial histone code by semi-supervised biclustering

**DOI:** 10.1186/1471-2164-13-301

**Published:** 2012-07-03

**Authors:** Li Teng, Kai Tan

**Affiliations:** 1 Department of Internal Medicine, University of Iowa, Iowa City, IA, USA; 2 Department of Biomedical Engineering, University of Iowa, Iowa City, IA, USA

**Keywords:** Epigenetics, Enhancers, Semi-supervised learning, Biclustering, ChIP, Mass spectrometry

## Abstract

**Background:**

Combinatorial histone modification is an important epigenetic mechanism for regulating chromatin state and gene expression. Given the rapid accumulation of genome-wide histone modification maps, there is a pressing need for computational methods capable of joint analysis of multiple maps to reveal combinatorial modification patterns.

**Results:**

We present the Semi-Supervised Coherent and Shifted Bicluster Identification algorithm (SS-CoSBI). It uses prior knowledge of combinatorial histone modifications to guide the biclustering process. Specifically, co-occurrence frequencies of histone modifications characterized by mass spectrometry are used as probabilistic priors to adjust the similarity measure in the biclustering process. Using a high-quality set of transcriptional enhancers and associated histone marks, we demonstrate that SS-CoSBI outperforms its predecessor by finding histone modification and genomic locus biclusters with higher enrichment of enhancers. We apply SS-CoSBI to identify multiple cell-type-specific combinatorial histone modification states associated with human enhancers. We show enhancer histone modification states are correlated with the expression of nearby genes. Further, we find that enhancers with the histone mark H3K4me1 have higher levels of DNA methylation and decreased expression of nearby genes, suggesting a functional interplay between H3K4me1 and DNA methylation that can modulate enhancer activities.

**Conclusions:**

The analysis presented here provides a systematic characterization of combinatorial histone codes of enhancers across three human cell types using a novel semi-supervised biclustering algorithm. As epigenomic maps accumulate, SS-CoSBI will become increasingly useful for understanding combinatorial chromatin modifications by taking advantage of existing knowledge.

**Availability and implementation:**

SS-CoSBI is implemented in C. The source code is freely available at http://www.healthcare.uiowa.edu/labs/tan/SS-CoSBI.gz.

## Background

Covalent modification of histone tails is a major epigenetic mechanism. Furthermore, multiple intra-nucleosomal or inter-nucleosomal histone modifications are frequently observed within the same genomic loci. The histone code hypothesis postulates that multiple histone modifications act in a combinatorial fashion to specify distinct chromatin states, which in turn regulate gene activities [1,2]. To completely characterize the histone code is a major goal of epigenetics research.

To date, Chromatin Immunoprecipitation coupled with microarray chip (ChIP-Chip) or deep sequencing (ChIP-Seq) is the predominant experimental technology for obtaining genome-wide maps of histone modifications. However, ChIP-based technologies have inherent resolution limit given the fragmentation limit of chromatin DNA [3]. Recently, mass spectrometry (MS) has been applied to effectively characterize and quantitate combinatorial histone codes within the same histone tail [4]. Generally speaking, the MS-based approach is not yet amenable to high-throughput analysis though progress has been made constantly. Given their higher resolution and more quantitative nature, combinatorial histone modifications obtained from MS-based studies provide valuable complementary information to genome-wide combinatorial histone modification analysis based on ChIP technologies.

Using ChIP-based and MS-based technologies, characteristic histone modification combinations have been observed at various functional DNA elements [1,5], such as the combination of H3K4me3 and H3K27me3 at transcriptionally poised gene promoters [6]. Collectively, these observations provide strong support to the histone code hypothesis and suggest that a diverse array of histone modification combinations is associated with a variety of DNA elements [2,4,7].

Computational methods have been developed to reveal histone code hidden in global histone modification maps. Hon et al. [8] developed ChromaSig to find histone modification “motifs” that are repeated across the genome. Jascheck and Tanay [9] proposed a spatial clustering algorithm to identify sets of common patterns defined over contiguous genomic regions. Ernst and Kellis [10] introduced an HMM-based algorithm to segment the epigenome into regions with characteristic histone mark combinations. Unlike above-mentioned algorithms that aim to identify combinatorial patterns involving all histone marks in the input data, Ucar et al. [11] proposed a biclustering-based algorithm, the COherent and Shifted Bicluster Identification algorithm (CoSBI), which comprehensively searches for combinatorial patterns involving only subsets of histone marks. Given that many combinatorial patterns only involve a few chromatin modifications [5], CoSBI is better suited for identifying subsets of re-occurring modifications given a compendium of histone modification maps. Because it focuses on subsets of most correlated patterns, the resulting patterns are more precise.

The rapid accumulation of global histone modification maps represents an invaluable body of knowledge, which from a computational perspective, can be leveraged to guide a clustering algorithm, i.e. providing a limited form of supervision. Such an approach is known as semi-supervised learning in the machine learning field [12,13]. Traditionally, machine learning has been studied either in the unsupervised paradigm (e.g., clustering) where all the data are unlabelled, or in the supervised paradigm (e.g., classification) where all the data are labelled. The goal of semi-supervised learning is to understand how combining labelled and unlabelled data may change the learning behavior, and design algorithms that take advantage of such a combination. Semi-supervised learning is of great interest because it can use readily available unlabelled data to improve supervised learning tasks when labelled data are scarce or expensive.

Here, we describe a semi-supervised version of the CoSBI algorithm (SS-CoSBI), which incorporates existing knowledge of histone codes into the learning process. Specifically, combinatorial histone modifications observed using tandem MS is used as prior probabilities of histone mark co-occurrence and incorporated into the biclustering process.

Using a set of known transcriptional enhancers and histone modifications characterized by both MS and ChIP-Seq protocol, we demonstrate that SS-CoSBI outperforms its predecessor by finding biclusters with higher enrichment of enhancers. We apply SS-CoSBI to enhancers in three human cell types, B, T and embryonic stem (ES) cells. Our analysis reveals cell-type-specific preferences of histone modification states associated with enhancers. We found that genes near enhancers with the same histone modification states have highly correlated expression between B and T cells but not ES cells, suggesting that histone modification states mostly potentiate enhancers but ultimately it is specific transcription factors at enhancers that determine their regulatory outcome. Finally, by overlapping enhancer histone modification states with DNA methylome data, we found that at ES cell enhancers H3K4me1 significantly correlates with a higher level of DNA methylation and reduced expression of nearby genes, suggesting that different combinations of histone modifications and DNA methylation at enhancers could lead to distinct regulatory consequences.

## Results

### Derivation of prior probabilities of histone mark co-occurrence using mass spectrometry data

**Figure 1:**
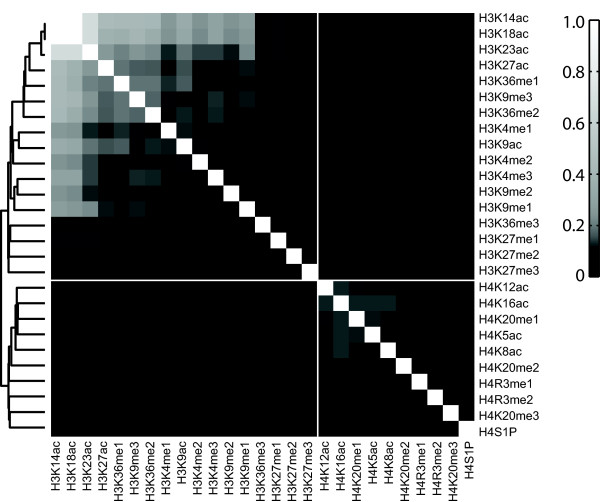
**Co-occurrence frequency matrix of 27 histone modifications determined by mass spectrometry experiments.** Histone modifications located within the same histone tail were identified by mass spectrometry. Raw frequency counts of histone modification pairs were normalized by the maximal observed co-occurrence count of histone modification pairs. Normalized matrix was clustered using hierarchical clustering with complete linkage; Pearson correlation of the normalized co-occurrence counts was used as the similarity measure. The block on the upper left corner contains histone 3 modifications and the block on the bottom right corner contains histone 4 modifications.

We compiled a list of 362 unique combinatorial histone modifications involving 27 modifications of histones H3 and H4 in human cells from published literature [14-17] (see Additional file 1: Table S1 for the 362 combinatorial histone modifications). Each combinatorial modification is observed in a single histone tail by tandem mass spectrometry. Using this list, we generated a 27 × 27 co-occurrence matrix based on the MS data. To construct the co-occurrence matrix, the raw counts of histone mark pairs were normalized by the count of the most frequent pair among all histone modification pairs. Figure 1 shows a hierarchical clustering on the rows of the co-occurrence matrix. The two apparent blocks in the dendrogram correspond to modifications within histone 3 and 4 tails, respectively. As more MS-based histone modification data becomes available, the co-occurrence matrix will become more accurate and comprehensive.

In the semi-supervised biclustering algorithm proposed here, the co-occurrence matrix derived from MS data is used as prior co-occurrence probabilities of histone modifications. In the rest of the paper, we used the 27 × 27 co-occurrence matrix defined by MS data in different ways depending on the histone modifications studied. In the section “Use of prior knowledge improved the quality of identified biclusters”, we constructed a 39 × 39 prior co-occurrence matrix by adding zeros for histone modifications not covered in the MS data. In the section “Application of semi-supervised CoSBI to enhancers in three human cell types”, we extracted a 5 × 5 submatrix covering the 5 histone modifications known to be associated with enhancers (H3K4me1, H3K4me2, H3K4me3, H3K9ac and H3K27ac).

### Use of prior knowledge improves the quality of identified biclusters

We compared the performance of SS-CoSBI and CoSBI in terms of their abilities to identify coherent biclusters from an input Genomic locus x Chromatin modification x Position in signal peak (GCP) matrix (see Methods) consisting of functional DNA elements and random genomic loci. Based on the histone code hypothesis, we expect genomic loci that share functionality to have a common chromatin modification signature. In contrast, random loci would lack a consistent signature. Given this assumption, an effective algorithm should be able to group together genomic loci of the same functional class along with their signature chromatin modifications. For comparison purpose, we used the dataset presented in [11]. It contains 213 high-confidence transcriptional enhancers, 213 random genomic loci, and the associated signals of 39 histone marks in human T cells.

Both CoSBI and SS-CosBI have four parameters: *min*_*g*_, *min*_*s*_, *alpha*, and *beta*. The first two parameters are the minimum numbers of genomic loci and histone modifications in the sought biclusters, respectively. For this benchmarking analysis, we set *min*_*g*_ to 43 (10% of the input data) and *min*_*s*_ to 3 to be consistent with the previous study using CoSBI. The other two parameters, *alpha* and *beta*, together determine the overall coherency of identified biclusters (see Methods). To compare the performance of the two algorithms, we ran both algorithms using *alpha* ranging from 0.65 to 0.8 with a step-size of 0.01 and *beta* ranging from 0.6 to 0.8 with a step-size of 0.01, resulting in 300 parameter combinations for each algorithm. The other two parameters, which determine the minimum size of the biclusters, were kept the same for both algorithms.

**Figure 2:**
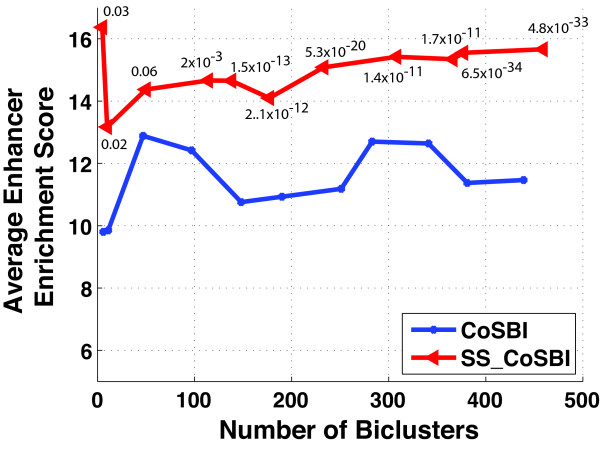
**Performance comparison of the original CoSBI and semi-supervised CoSBI (SS-CoSBI) algorithms across a range of*alpha*and*beta*parameter settings.** X-axis, bicluster sets of different sizes generated by the two algorithms using different parameter settings. Y-axis, the enhancer enrichment scores for each bicluster set. First, a hypergeometric p-value was computed for each bicluster in a set. Then, the enrichment score for each set was computed as the minus logarithm (base 2) of the average hypergeometric p-values of each bicluster set. Values around the SS-CoSBI curve are KS test p-values of enrichment score distributions. Each pair of bicluster sets of the same or similar size generated by CoSBI and SS-CoSBI was compared.

To evaluate the quality of the resulting biclusters we determined the functional ‘purity’ of the identified biclusters, i.e., the fraction of enhancers present in each bicluster. To do so, we calculated a hypergeometric p-value of enhancer enrichment for each bicluster. Figure 2 shows a comparison of the average significance of enhancer enrichment among bicluster sets generated by the two algorithms using a range of parameter combinations. SS-CoSBI outperformed CoSBI in terms of enhancer enrichment over the entire range of parameter settings tested. To assess the statistical significance of the enrichment p-value difference, we did a Kolmogorov-Smirnov test for each pair of bicluster sets of the same or similar size generated by the two algorithms. Again, in all cases the difference in enhancer enrichment was significant (Figure 2). This comparison demonstrates that incorporation of prior knowledge in the learning process increases the purity of biclusters generated by SS-CoSBI.

### Application of semi-supervised CoSBI to enhancers in three human cell types

Accumulating evidence suggests that histone modification patterns at enhancers are complex [7,18-22]. To gain insight into the histone code associated with enhancers, we applied SS-CoSBI to three human cell types, B, T and ES cells. For each cell type, we identified a set of high-confidence enhancers as p300 ChIP-Seq peaks that are distal from gene promoters (> 2.5 k bp). The resulting sets of enhancers are mostly cell-type-specific (see Additional file 2: Figure S1). For histone modification, we studied the following five marks, H3K4me1, H3K4me2, H3K4me3, H3K9ac and H3K27ac, because various subsets of these marks have been used as signatures for enhancers in previous studies [18,19,23,24].

**Figure 3:**
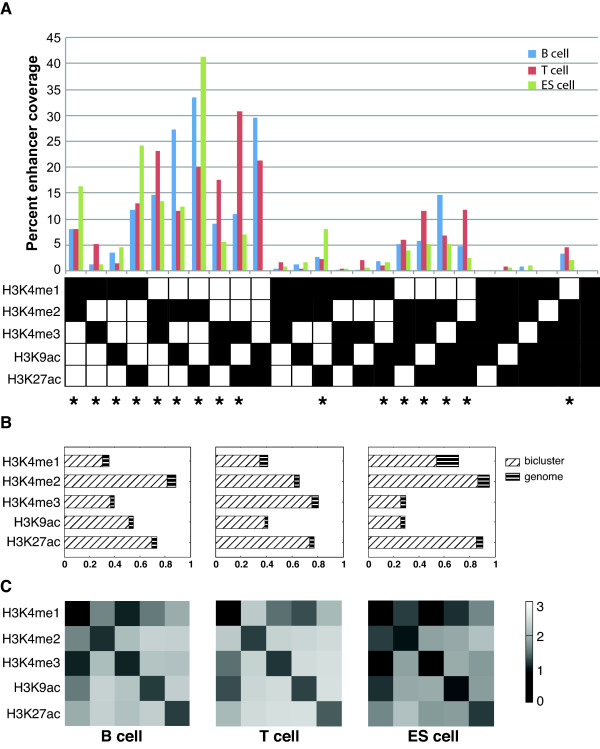
**Statistics of enhancer biclusters.A)** Enhancer coverage of all 26 possible histone states involving at least two histone modification marks. Y-axis, percentages of enhancers in biclusters that are associated with a given histone state. Histone marks of each state is indicated at the bottom of the histogram. Each column represents a histone state with the filled square indicating the presence of a histone mark in the state and empty square otherwise. Asterisk, histone states shared by all three cell types and cover at least 1% of the enhancers. **B)** Histone mark occurrence frequencies in the genome and in the 16 common biclusters shared among three cell types. Frequencies are indicated by the length of the bar for genomic and bicluster categories separately. **C)** Normalized co-occurrence frequencies of histone mark pairs in the 16 common biclusters shared by all three cell types. Shown are log ratios (base 10) of observed versus expected frequencies of histone mark pairs. Expected frequencies are calculated using genomic frequencies of individual histone marks.

For this genome-wide analysis, we set *min*_*s*_ to two since we are interested in combinatorial patterns involving at least two histone modifications. With five histone modifications, the total number of combinations containing at least two histone modifications is 26. These combinations represent possible histone modification states at enhancers. After biclustering, an observed histone state was defined as the set of histone marks associated with a particular bicluster. In the rest of the paper, we used a set of codes to represent these states in a compact and meaningful way. For instance, the code M12A9 represents the histone mark combination of H3K4me1, H3K4me2, and H3K9Ac. For comparison purpose, we ran SS-CoSBI with parameter combinations (*min*_*g*_, *alpha*, and *beta*) to obtain the same number of biclusters (n = 22) and enhancer coverage (60%) for all three cell types, respectively. In the resulting set of biclusters, enhancer coverage ranges from 0.26% to 41.40% (Figure 3A, Additional file 3: Table S6). Sixteen of the 22 histone states (labelled with an asterisk in Figure 3A) are shared by all three cell types and have enhancer coverage greater than 1%.

To compare the identified chromatin states of enhancers with that of promoters, we performed the same biclustering experiment on promoters, using 3000 most highly expressed genes in each of the three cell types. To be consistent with what we have done for enhancers, we used a 5 k bp window for promoters (4.5 k bp upstream and 0.5 k bp downstream of RefSeq TSS). The same parameter setting of SS-CoSBI was used as in the enhancer analysis. Additional file 2: Figure S3 shows the percentage of promoters containing each combinatorial histone modification pattern. To compare the overall similarity of chromatin states between enhancers and promoters, we calculated Pearson correlation of chromatin state distribution between the two types of DNA elements, using the 16 chromatin states that are common to all three cell types and have enhancer coverage larger than 1% in the enhancer analysis. The correlation coefficients are 0.43, 0.23, and 0.12 for B, T, and ES cell, respectively. All three correlations are not statistically significant using the theoretical distribution of Pearson correlation coefficient (p = 0.1, 0.4, 0.7 respectively). Therefore, we conclude that the overall chromatin state distributions of our enhancer biclusters are distinct from promoters.

Recently, Ernst et al. [20] applied a multivariate hidden Markov model (HMM) to ChIP-Seq data of 9 histone modifications to infer chromatin modification states associated with various types of functional DNA elements, including enhancers. We compared the sets of enhancer biclusters from this study and enhancers identified using HMM in human ES and B cells. Among the 22 biclusters for both ES and B cells, 10 and 16 of them significantly overlap with the set of enhancers identified by Ernst et al., respectively Additional file 1: Table S3 and Table S4), suggesting that both approaches can reveal histone states associated with enhancers. On the other hand, for those biclusters that do not have significant overlap with the Ernst et al. set, they could mean that these are enhancer histone states that were missed by the HMM approach, highlighting the strength of the biclustering approach.

### Enhancers exhibit cell-type-specific histone states

As shown in Figure 3A, enhancers in different cell types have characteristic distributions of histone modification states. For instance, the top three histone states are (M2A27, A927, M2A9), (M3A27, M23, A927) and (M2A27, M1A27, M12) for B, T, and ES cells, respectively. Furthermore, the occurrence frequencies of individual histone marks and pairs of histone marks are also different among different cell types as shown in Figure 3B and C, respectively. For B and ES cells, the most frequent mark is H3K4me2. For T cell, the most frequent mark is H3K4me3. Clearly, occurrence frequencies of histone marks in enhancer biclusters are different from their genomic frequencies (Figure 3B), consistent with the notion that these histone marks are signatures associated with enhancers. With regard to pairwise occurrence frequency, the most frequent pairs are (H3K4me2, H3K9ac), (H3K9ac, H3K27ac), and (H3K4me2, H3K27ac) for B, T, and ES cells, respectively. For each of these histone mark pairs, their co-occurrence in enhancer bicluster is 100 times more frequent than what is expected based on their individual genome-wide frequencies, consistent with the combinatorial nature of these histone codes for enhancers. Many histone modification enzymes are recruited to enhancers by transcription factors. Therefore, the observed cell-type-specific histone codes of enhancers may reflect the activities of cell-type-specific TFs at enhancers.

### Correlation between enhancer histone code and gene expression

**Figure 4:**
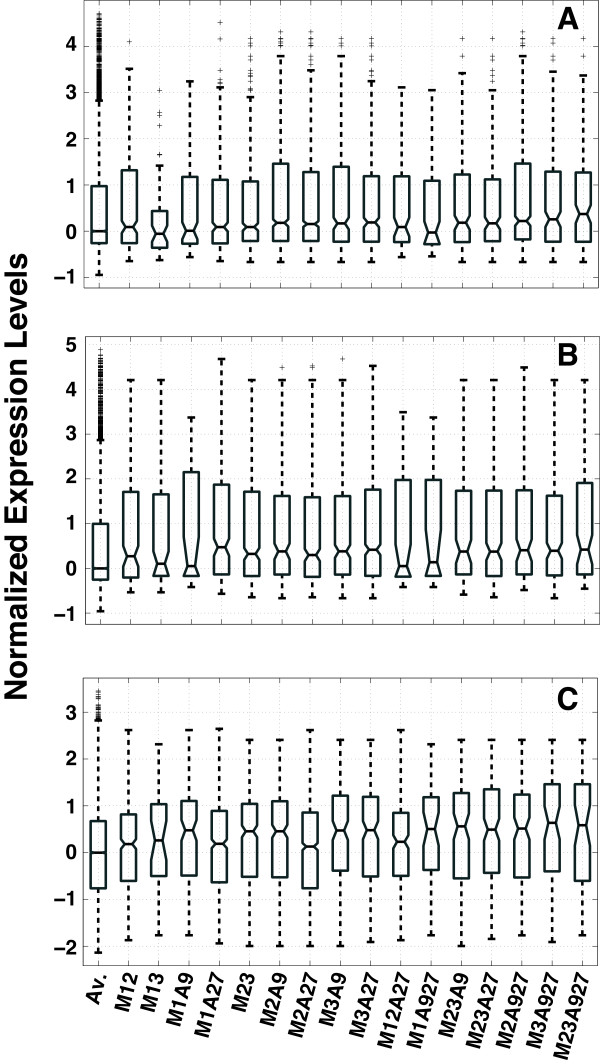
**Boxplots of expression levels of genes closest to each of the 16 common biclusters shared by all three cell types. A)** B cell. **B)** T cell. **C)** ES cell. Av., mean normalized gene expression level of all genes in each cell type. Enhancer histone modification states are indicated at the bottom.

To better understand the functional difference of the enhancer classes defined based on their histone modification states, we asked if there is a correlation between enhancer histone modification state and expression level of their target genes. Since identifying enhancer targets is an unsolved problem, following common practice, we used the closest RefSeq gene as the target of an enhancer. For each enhancer bicluster, we obtained the expression levels of all genes closest to the enhancers in the bicluster. To allow comparison of gene expression across biclusters and cell types, we applied z-score transformation of gene expression data followed by median normalization on all available genes for each cell type. Figure 4 shows the normalized expression levels associated with different enhancer biclusters.

Overall, expression levels of target genes of all biclusters are equal to or higher than the genome average, which is consistent with the role of enhancers. Across all three cell types, median expression levels of target genes vary among different enhancer biclusters. Furthermore, the variations are highly correlated between B and T cells (Pearson correlation = 0.74, p-value = 1.1 × 10^-3^). However, we only observed moderate correlations between ES cells and T or B cells. The Pearson correlations are 0.47 (p-value = 0.07) and 0.30 (p-value = 0.27), respectively.

In summary, the above results demonstrate that multiple classes of enhancers can be defined by their histone modification states, which in turn may influence the activities of the enhancers as judged by the expression levels of their nearby genes. On the other hand, although histone modification states of enhancers can modulate their activities, it is clearly not the only determinant as indicated by the lower correlation between lymphocytes and ES cells. Other factors, such as TFs and nucleosome remodelling proteins also play an important role in modulating enhancer activity.

### Two classes of enhancers defined by H3K4me1 and DNA methylation level in ES cells

Besides histone modifications, DNA methylation is also found at enhancers [25-28]. However, how the two types of epigenetic marks interact at enhancers is poorly understood [29-31]. To address this issue, we first examined DNA methylation levels of ES cell enhancers in the biclusters, using a recently published single-base-resolution methylome map of human ES cells [32]. We could not study B and T enhancers since the same type of data is not available for these two cell types.

**Figure 5:**
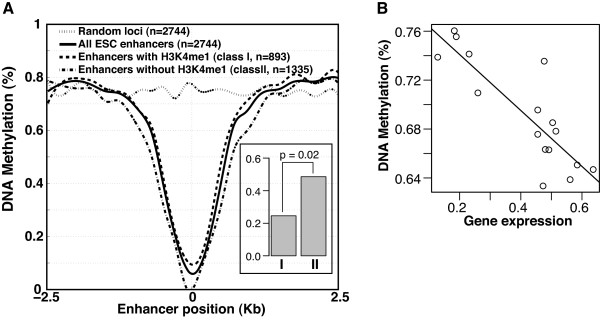
**Two classes of enhancers defined by the presence and absence of H3K4me1 in their histone states and their DNA methylation levels. A)** DNA methylation profiles of two bicluster classes and random genomic loci. Inset, median expression levels of genes next to enhancers of the two classes. P-value is based on one-tailed *t*-test. **B)** Correlation between bicluster DNA methylation level and expression level of nearby genes.

Consistent with a recent finding about enhancers in mouse [27], we found a decreased DNA methylation level at enhancers compared to random genomic loci (Figure 5A). In addition, average DNA methylation level of enhancers is negatively correlated with expression levels of nearby genes (Pearson correlation = -0.85, p-value = 3.1 × 10^-5^, Figure 5B), consistent with the notion of DNA methylation being a repressive marker of transcription.

Next, to elucidate the interplay between DNA methylation and histone code at ES cell enhancers, we compared DNA methylation levels of different enhancer biclusters. Strikingly, we found that the different enhancer biclusters can be grouped into two classes based on their DNA methylation levels (Figure 5A). Enhancers with the H3K4me1 mark (termed class I here) tend to have significantly higher DNA methylation levels than those without the H3K4me1 mark (class II), although for both classes DNA methylation levels are lower than random genomic loci (Figure 5A, Table 1). Furthermore, genes closest to class I enhancers have significantly lower expression levels compared to those closest to class II enhancers (Figure 5A inset). Next, we examined the functional annotations of the genes near the two classes of enhancers using the GREAT software [33]. At a FDR of 0.05, we found that genes near class II enhancers are highly enriched for stem cell development and maintenance and other early developmental processes. In contrast, genes near class I enhancers have fewer enriched terms that are involved in non-developmental processes (Additional file 4: Table S7).

**Table 1:** DNA methylation levels of two classes of enhancers in ES cells

**Bicluster ID**	**Enhancer class**	**# enhancers**	**Median DNA methylation**	**P-value**
M12	I	447	0.76	1
M13	I	33	0.71	0.08
M1A9	I	127	0.74	1
M1A27	I	664	0.76	1
M12A27	I	222	0.74	0.29
M1A927	I	47	0.60	0.03
M23	II	371	0.67	5.1e-40
M2A9	II	342	0.69	4.8e-18
M2A27	II	1136	0.74	2.6e-8
M3A9	II	153	0.63	5.7e-25
M3A27	II	193	0.66	4.8e-27
M23A9	II	109	0.64	3.3e-19
M23A27	II	144	0.66	9.5e-21
M2A927	II	140	0.68	7.0e-14
M3A927	II	69	0.65	5.3e-12
M23A927	II	56	0.65	2.2e-9

Enhancer class is defined by the presence (class I) or absence (class II) of the histone mark H3K4me1 in their histone states. To compute the median DNA methylation level of a given bicluster, we first calculated a methylation level for each CpG cytosine with at least 10 sequencing reads as the ratio of the number of methylated reads versus the total number of reads, using the single-base-resolution methylome map by Lister et al. [32]. Next, for each enhancer region, an average DNA methylation level of all CpG cytosines was calculated. Finally, a median DNA methylation level of all enhancers in a bicluster was then calculated. The median DNA methylation level of all 2744 ES cell enhancers used in this study is 0.76. We compared the DNA methylation levels of each set of bicluster enhancers to that of all ES cell enhancers used in this study using the Komolgonov-Smirnov test. P-values are corrected for multiple testing using the Bonferroni method.

Recently, Pekowska et al. [34] demonstrated that reduced enhancer activity is associated with the loss of H3K4me3 and concurrent increase in H3K4me1 in T cells. Our result above is consistent with their observation with regard to the relationship between different H3K4 methylation marks and enhancer activity. Furthermore, beyond histone methylation, our data suggest that there is an intricate interplay between different H3K4 methylation marks and DNA methylation, which in turn can modulate the activities of enhancers. Enhancers that are marked by H3K4me2 and/or H3K4me3 but lack H3K4me1 appear to have lower DNA methylation levels and higher activities, and are located near genes important for early developmental processes. In contrast, enhancers marked by H3K4me1 tend to have higher DNA methylation levels and lower activities, and are located near genes that are not involved in early development.

## Discussion and Conclusions

ChIP-Seq and tandem MS are the two dominant technologies for mapping combinatorial histone modifications. Both technologies have pros and cons. MS-based data is less noisy and more quantitative. However, it does not provide information about the histone modification status at individual genomic loci. ChIP-Seq-based data provides information for all genomic loci but it is noisier and has a lower resolution (i.e. typically several hundred nucleotides). Here, by incorporating MS-based combinatorial histone code with ChIP-Seq data in a semi-supervised biclustering framework, we not only improve the accuracy of discovering combinatorial histone codes but also extend the utility of MS-based data by connecting them back to specific loci across the genome.

Here we used MS-based data to learn the prior probabilities of histone mark co-occurrence. However, in principle, any prior knowledge about combinatorial histone modifications can be used to learn priors, including knowledge obtained using biochemical assays and published ChIP-Chip/Seq data. The latter type of data is particularly useful given the rapid and steady accumulation of ChIP-Seq-based epigenomic maps under various conditions. However, care must be taken to avoid using the same ChIP-Chip/Seq data in a circular fashion.

The analysis presented here provides a systematic characterization of combinatorial histone codes of enhancers across three cell types. Most previous studies of enhancer histone modification focused only on a subset of these five histone marks. Among the five histone marks, H3K4me1 is the first and thus most widely used surrogate for enhancers [24]. However, more recent studies have demonstrated that histone modifications at enhancers are complicated, both in the number of different modifications and their combinations. Besides H3K4me1, H3K4me2, H3K9Ac and H3K27Ac have been shown to mark enhancers [19,21,35]. More recently, even H3K4me3, which is regarded by many not an enhancer marker, has been shown to mark active enhancers [33]. This finding is consistent with the Wang et al. analysis of T cell enhancers, which also demonstrated an enrichment of H3K4me3 at enhancers [7]. A possible explanation for this apparent discrepancy is that H3K4me3 activity is cell type specific. Clearly, more studies are needed to resolve this issue. Nevertheless, in this study, we took a step further by using five histone marks reported to be associated with enhancers in various studies and focusing on their combinations, our result highlights the complexity of histone modifications at enhancers and possibly other functional DNA elements. For instance, we found enhancers that have combinations of other histone marks but H3K4me1. Given that H3K4me1 is frequently observed at enhancer, this was initially a bit surprising. Our additional analysis indicates these enhancers are enriched for cis-regulatory modules (PreMod modules, Additional file 1: Table S5) and some of these overlap with enhancers predicted in Ernst et al. (Additional file 1: Tables S3, S4). Therefore, they are likely bona fide enhancers.

In the future, analysis of multiple epigenetic marks and comparing them across different cell types and conditions will greatly improve our understanding of how epigenetic factors affect gene expression. In addition, the biclustering framework of SS-CoSBI can also be applied to study combinatorial binding of multiple transcription factors in enhancers.

## Methods

### Data source and processing

#### Combinatorial histone modifications identified by tandem mass spectrometry

Histone modifications within a single histone tail identified by tandem mass spectrometry experiments were compiled from published literature [14-17]. This set contains 362 histone modification combinations covering 27 histone marks, including 17 methylations (H3K4me1, H3K4me2, H3K4me3, H3K9me1, H3K9me2, H3K9me3, H3K27me1, H3K27me2, H3K27me3, H3K36me1, H3K36me2, H3K36me3, H4K20me1, H4K20me2, H4K20me3, H4R3me1, and H4R3me2), 9 acetylations (H3K9ac, H3K14ac, H3K18ac, H3K23ac, H3K27ac, H4K5ac, H4K8ac, H4K12ac, and H4K16ac) and 1 phosphorylation (H4S1P). The set of combinatorial histone marks is shown in Additional file 1: Table S1.

#### Histone modification ChIP-seq data

Raw ChIP-Seq sequence reads for human H1 ES cell and CD4+ T cell were obtained from NCBI Short Read Archive from references [7,36] (T cell) and [37] (ES cell). For B cell, we downloaded the data from the ENCODE project website [38]. For each histone modification, there are multiple replicate datasets for H1 ES cell. We used the one with largest number of mapped reads.

We aligned the ChIP-Seq reads to the human genome (build hg18) using Bowtie [39]. To control for sequencing depth of each data set, we followed the approach by Hon et al. [8]. First, the genome was partitioned into 200 bp bins. We then normalized the number of reads in each bin with a sigmoid function,

(1) bi{h}'=11+e−bi{h}−medianb{h}/stdb{h}

where b_i{h}_ is the read count of bin *i* in histone modification *h*, and *median*(*b*_{h}_) and *std*(*b*_{*h*}_) are the median and standard deviation of read counts of all 200 bp bins in the genome for histone modification *h*, respectively. We excluded bins that have 0 or 1 read.

#### Transcriptional enhancers

P300 is a transcriptional co-activator that is frequently observed at enhancers. P300 ChIP-Seq data were available for all three cell types. To avoid confusion with promoters, we selected distal p300 binding peaks (≥ 2.5 k bp away from known RefSeq transcription start sites (TSS)) as known enhancers [40]. Defining the complete list of TSS itself is a challenging task. We chose the RefSeq annotations because it is most likely the most comprehensive and reliable set to date. To ensure that selected p300 peaks contain histone modification marks under study, distal p300 peaks that did not overlap any histone modification peaks were excluded. In total, 14.2%, 11.7%, and 7.4% of the peaks were removed for B, T, and ES cells, respectively. This resulted in 4310, 2097, and 2744 distal p300 peaks for B, T, and ES cells, respectively. The vast majority of the resulting p300 peaks have more than one histone marks (see Additional file 1: Table S2). For the benchmarking experiment, we further filtered distal p300 peaks in T cell to include only those that overlap with at least one computationally predicted enhancer in the PReMod database [41]. This procedure produced 213 high-confidence enhancers. We then extracted normalized ChIP-Seq signals of each histone modification within the 5 k bp region centered on each distal p300 peak.

#### Gene expression data

We compiled a compendium of gene expression profiles for the three cell types under study from [42,43]. The compendium covers 23975 RefSeq genes for B and T cells, and 26173 RefSeq genes for ES cell, respectively.

#### Construction of GCP matrix

The input to both CoSBI and SS-CoSBI (in addition to the prior co-occurrence probability matrix) is a 3-dimensional matrix of pre-processed histone modification ChIP-Seq data. We termed the three dimensions as Genomic locus, Chromatin modification, and Position in a signal peak. Therefore, the matrix is abbreviated as a **GCP** matrix. For the benchmarking experiment, dimensions of the matrix are 426 (number of high confidence enhancers and random genomic loci in T cell) × 39 (number of histone modifications) ×25 (number of signals per genomic locus). For the genome-wide enhancer analysis, dimensions of the matrix are N (number of enhancers with at least one histone modification peak) ×5 (number of histone modifications) ×25 (number of signals per genomic locus).

### Semi-supervised biclustering

In [11], the authors proposed an algorithm, CoSBI, to exhaustively search for maximal coherent biclusters which show combinatorial histone modification patterns that frequently recur in an epigenome and exhibit similar signals. The algorithm takes a three-dimensional (3D) matrix, the **GCP** matrix, as the input data. The algorithm has four parameters, *alphabetamin*_g_ and *min*_s_. *Alpha* and *beta* set the minimum coherency threshold across genomic loci and histone marks, respectively. *min*_g_ and *min*_s_ set the minimum numbers of genomic loci and histone modifications in a bicluster, respectively. A resulting coherent bicluster is a sub-matrix of **GCP**, which covers the signals of a subset of genomic loci in a subset of histone marks.

Here we present the Semi-Supervised CoSBI (SS-CoSBI) algorithm, which incorporates prior knowledge of combinatorial histone modifications into the CoSBI framework. First, we generate the prior probability matrix **P** of histone mark co-occurrence based on the pairwise co-occurrence frequencies observed in a compendium of MS experiments. Then we incorporate **P** in the learning process by using it to adjust the similarity measure among histone modification signals.

In CoSBI, maximum cross correlation between two signal vectors is used as the similarity measure. With m histone modifications there is an m × m similarity matrix **C** representing the correlations among the set of histone modifications for each genomic locus. We first convert the correlation matrix **C** to a distance measure matrix **D**.

(2) $$D_{\mathrm{ij}}=1-C_{\mathrm{ij}}$$

Where *C*_*ij*_ is the maximal cross correlation between histone mark *i* and *j*. Then we adjust the distance matrix by incorporating MS-derived prior co-occurrence probabilities,

(3) Dij'=(1−Pij)×Dij

where *P*_*ij*_ is the prior probability of co-occurrence between histone mark *i* and *j*. If there is no prior knowledge about the co-occurrence of histone mark *i* and *j*, then *P*_*ij*_ equals to zero and *D*_*ij*_ stays unchanged. If there is prior knowledge about the co-occurrence of histone mark *i* and *j*, the higher the prior probability is, the more the distance is decreased. To ensure that the triangular inequality of a distance measure is maintained, we use the Floyd-Warshall algorithm (all pair shortest paths algorithm) [44] to update the distance matrix. Finally, we convert the distance matrix back to the correlation matrix **C′**.

(4) $$C_{\mathrm{ij}}\prime =1\_\text{all}\_\text{pair}\_\text{shortest}\_\text{path}\;\left({\text{D}}_{{}_{\mathrm{ij}}}\prime \right)$$

where *C*_*ij*_′ is the updated cross correlation between histone mark *i* and *j*. SS-CoSBI takes two input data, the **GCP** matrix representing the histone modification data and the **P** matrix representing the prior probability matrix of histone co-occurrence.

There are two main steps of the algorithm to identify biclusters. In the first step, all maximal coherent histone modification sets for each genomic locus are identified. A maximal coherent histone modification set is a clique of histone modifications where any two members have a similarity higher than the threshold *beta*. Cross correlation was used in this study as the similarity measure. An inverted list is then constructed based on the maximal coherent histone modification sets for each genomic locus. The final step is to identify the maximal coherent genomic locus set for every combination of histone modifications that satisfy the minimal similarity threshold *alpha,* minimal number of genomic loci *min*_g_, and minimal number of histone modifications *min*_s_ Several searching and pruning strategies on the set enumeration tree were implemented for both two steps that enable the algorithm to find all coherent biclusters efficiently. See Additional file 5: Figure S2 for the pseudo code of the SS-CoSBI algorithm.

### Merge overlapping biclusters

Non-exclusive clustering algorithms often generate overlapping clusters, sometimes due to noisy input data. Overlapping biclusters can share common genomic loci, or common histone modifications, or both. When two biclusters share a large portion of genomic loci or histone modifications they should be merged to generate a larger and less redundant bicluster. Here, we propose the following procedure to merge overlapping biclusters. For two biclusters Bi_1_ and Bi_2_, we define an overlap ratio *r* to quantify the degree of overlap between the two biclusters.

(5) $$r=\frac{n\prime \times m\prime }{N\times M}$$

where *N* and *M* are total numbers of genomic loci and histone modifications covered by the two biclusters, and *n*′ and m′ are numbers of shared genomic loci and histone modifications by the two biclusters, respectively. The overlap ratio takes into account the sizes of both biclusters and the size of the overlapping part. Merging is carried out using a scheme similar to hierarchical agglomerative clustering with complete linkage. An overlapping ratio matrix is constructed for all bicluster pairs. In each iteration, two biclusters having the largest overlapping ratio are merged and the overlapping ratio matrix is updated. The merging stops when the largest overlapping ratio is below some threshold. We used 0.75 as the threshold in this study.

## Competing interests

The authors declare that they have no competing interests.

## Authors’ contributions

LT and KT conceived and designed the study. LT performed the experiments. LT and KT analyzed the data. LT and KT wrote the manuscript. Both authors have read and approved the final manuscript.

## Supplementary Material

Additional file 1 **Table S1.** List of 362 combinatorial histone modifications identified by tandem mass spectrometry [14-17]. **Table S2.** Number and fraction of distal p300 peaks that have only one histone modification peak. **Table S3.** Overlap between B cell enhancers from the biclusters in this study and those reported in Ernst et al. [20]. **Table S4.** Overlap between ES cell enhancers from the biclusters in this study and those reported in Ernst et al. [20]. **Table S5.** Overlap between enhancer biclusters that have more H3K4me1 mark and PReMod cis-regulatory modules. Click here for file

Additional file 2 **Figure S1.** Venn diagram of enhancers in three cell types used as inputs to the biclustering algorithms. **Figure S3.** Promoter coverage of all 26 possible histone states involving at least two histone modification marks. Click here for file

Additional file 3 **Table S6.** Histone states of enhancers as defined by biclustering in B, T, and ES cells. Click here for file

Additional file 4 **Table S7.** Gene ontology term enrichment among genes located near two classes of enhancers in ES cells. Click here for file

Additional file 5 **Figure S2.** Pseudo code for the semi-supervised CoSBI algorithm. Click here for file
