# Supplementary material for: Finding combinatorial histone code by semi-supervised biclustering
Source: BMC Genomics. 2012 Jul 3;13:301. doi: 10.1186/1471-2164-13-301 (PMC3443427; doi:10.1186/1471-2164-13-301)
Supplement: Additional file 1 — Table S1. List of 362 combinatorial histone modifications identified by tandem mass spectrometry [14-17]. Table S2. Number and fraction of distal p300 peaks that have only one histone modification peak. Table S3. Overlap between B cell enhancers from the biclusters in this study and those reported in Ernst et al. [20]. Table S4. Overlap between ES cell enhancers from the biclusters in this study and those reported in Ernst et al. [20]. Table S5. Overlap between enhancer biclusters that have more H3K4me1 mark and PReMod cis-regulatory modules. [file 1471-2164-13-301-S1.pdf]

Table S1. List of 362 combinatorial histone modifications identified by tandem mass spectrometry. Each row represents a set of histone modifications observed within the same single histone tail. Data were obtained from the following publications: [1-4]

Combinatorial histone modifications	Reference
H3K14ac,H3K18ac,H3K23ac,H3K27ac,H3K36me1,H3K9me3	LeRoy G. et al.
H3K14ac,H3K18ac,H3K23ac,H3K27ac,H3K36me2,H3K4me1,H3K9ac	LeRoy G. et al.
H3K14ac,H3K18ac,H3K23ac,H3K27ac,H3K36me2,H3K4me2,H3K9ac	LeRoy G. et al.
H3K14ac,H3K18ac,H3K23ac,H3K27ac,H3K36me2,H3K4me3,H3K9me2	LeRoy G. et al.
H3K14ac,H3K18ac,H3K23ac,H3K27ac,H3K36me2,H3K9me1	LeRoy G. et al.
H3K14ac,H3K18ac,H3K23ac,H3K27ac,H3K36me2,H3K9me2	LeRoy G. et al.
H3K14ac,H3K18ac,H3K23ac,H3K27ac,H3K36me3,H3K4me1	LeRoy G. et al.
H3K14ac,H3K18ac,H3K23ac,H3K27ac,H3K36me3,H3K4me1,H3K9ac	LeRoy G. et al.
H3K14ac,H3K18ac,H3K23ac,H3K27ac,H3K36me3,H3K9ac	LeRoy G. et al.
H3K14ac,H3K18ac,H3K23ac,H3K27ac,H3K36me3,H3K9me1	LeRoy G. et al.
H3K14ac,H3K18ac,H3K23ac,H3K27ac,H3K4me1,H3K9me3	LeRoy G. et al.
H3K14ac,H3K18ac,H3K23ac,H3K27me1,H3K36me1,H3K4me1,H3K9me2	Garcia B. et al.
H3K14ac,H3K18ac,H3K23ac,H3K27me1,H3K36me1,H3K9me1	LeRoy G. et al.
H3K14ac,H3K18ac,H3K23ac,H3K27me1,H3K36me2,H3K4me1	Garcia B. et al.
H3K14ac,H3K18ac,H3K23ac,H3K27me1,H3K36me2,H3K4me1,H3K9ac	LeRoy G. et al.
H3K14ac,H3K18ac,H3K23ac,H3K27me1,H3K36me2,H3K4me1,H3K9me1	Garcia B. et al.
H3K14ac,H3K18ac,H3K23ac,H3K27me1,H3K36me2,H3K4me1,H3K9me2	Garcia B. et al. LeRoy G. et al.
H3K14ac,H3K18ac,H3K23ac,H3K27me1,H3K36me2,H3K4me1,H3K9me3	LeRoy G. et al.
H3K14ac,H3K18ac,H3K23ac,H3K27me1,H3K36me2,H3K9me1	Garcia B. et al. LeRoy G. et al.
H3K14ac,H3K18ac,H3K23ac,H3K27me1,H3K36me2,H3K9me2	LeRoy G. et al.
H3K14ac,H3K18ac,H3K23ac,H3K27me1,H3K36me2,H3K9me3	Garcia B. et al. LeRoy G. et al.
H3K14ac,H3K18ac,H3K23ac,H3K27me1,H3K36me3,H3K4me1,H3K9me1	LeRoy G. et al.
H3K14ac,H3K18ac,H3K23ac,H3K27me1,H3K36me3,H3K4me1,H3K9me3	LeRoy G. et al.
H3K14ac,H3K18ac,H3K23ac,H3K27me1,H3K36me3,H3K4me2,H3K9me2	LeRoy G. et al.
H3K14ac,H3K18ac,H3K23ac,H3K27me1,H3K36me3,H3K4me2,H3K9me3	LeRoy G. et al.
H3K14ac,H3K18ac,H3K23ac,H3K27me1,H3K36me3,H3K4me3,H3K9ac	LeRoy G. et al.
H3K14ac,H3K18ac,H3K23ac,H3K27me1,H3K36me3,H3K4me3,H3K9me2	LeRoy G. et al.
H3K14ac,H3K18ac,H3K23ac,H3K27me1,H3K36me3,H3K9ac	LeRoy G. et al.
H3K14ac,H3K18ac,H3K23ac,H3K27me1,H3K36me3,H3K9me1	LeRoy G. et al.
H3K14ac,H3K18ac,H3K23ac,H3K27me1,H3K36me3,H3K9me3	LeRoy G. et al.
H3K14ac,H3K18ac,H3K23ac,H3K27me1,H3K4me2,H3K9me3	LeRoy G. et al.
H3K14ac,H3K18ac,H3K23ac,H3K27me1,H3K9me2	LeRoy G. et al.
H3K14ac,H3K18ac,H3K23ac,H3K27me2	LeRoy G. et al.
H3K14ac,H3K18ac,H3K23ac,H3K27me2,H3K36me1	Garcia B. et al.
H3K14ac,H3K18ac,H3K23ac,H3K27me2,H3K36me1,H3K4me1	Garcia B. et al.
H3K14ac,H3K18ac,H3K23ac,H3K27me2,H3K36me1,H3K4me1,H3K9ac	LeRoy G. et al.
H3K14ac,H3K18ac,H3K23ac,H3K27me2,H3K36me1,H3K4me1,H3K9me2	LeRoy G. et al.
H3K14ac,H3K18ac,H3K23ac,H3K27me2,H3K36me1,H3K4me1,H3K9me3	LeRoy G. et al.
H3K14ac,H3K18ac,H3K23ac,H3K27me2,H3K36me1,H3K4me3,H3K9me2	LeRoy G. et al.
H3K14ac,H3K18ac,H3K23ac,H3K27me2,H3K36me1,H3K9me1	Garcia B. et al.

H3K14ac,H3K18ac,H3K23ac,H3K27me2,H3K36me1,H3K9me2	Garcia B. et al. LeRoy G. et al.
H3K14ac,H3K18ac,H3K23ac,H3K27me2,H3K36me1,H3K9me3	Garcia B. et al. LeRoy G. et al.
H3K14ac,H3K18ac,H3K23ac,H3K27me2,H3K36me2,H3K4me1	Garcia B. et al.
H3K14ac,H3K18ac,H3K23ac,H3K27me2,H3K36me2,H3K4me1,H3K9ac	LeRoy G. et al.
H3K14ac,H3K18ac,H3K23ac,H3K27me2,H3K36me2,H3K4me1,H3K9me1	Garcia B. et al.
H3K14ac,H3K18ac,H3K23ac,H3K27me2,H3K36me2,H3K4me1,H3K9me2	Garcia B. et al. LeRoy G. et al.
H3K14ac,H3K18ac,H3K23ac,H3K27me2,H3K36me2,H3K4me1,H3K9me3	LeRoy G. et al.
H3K14ac,H3K18ac,H3K23ac,H3K27me2,H3K36me2,H3K4me2,H3K9ac	LeRoy G. et al.
H3K14ac,H3K18ac,H3K23ac,H3K27me2,H3K36me2,H3K4me2,H3K9me2	LeRoy G. et al.
H3K14ac,H3K18ac,H3K23ac,H3K27me2,H3K36me2,H3K4me2,H3K9me3	LeRoy G. et al.
H3K14ac,H3K18ac,H3K23ac,H3K27me2,H3K36me2,H3K4me3,H3K9me1	LeRoy G. et al.
H3K14ac,H3K18ac,H3K23ac,H3K27me2,H3K36me2,H3K4me3,H3K9me2	LeRoy G. et al.
H3K14ac,H3K18ac,H3K23ac,H3K27me2,H3K36me2,H3K9ac	LeRoy G. et al.
H3K14ac,H3K18ac,H3K23ac,H3K27me2,H3K36me2,H3K9me1	Garcia B. et al. LeRoy G. et al.
H3K14ac,H3K18ac,H3K23ac,H3K27me2,H3K36me2,H3K9me2	Garcia B. et al. LeRoy G. et al.
H3K14ac,H3K18ac,H3K23ac,H3K27me2,H3K36me2,H3K9me3	Garcia B. et al. LeRoy G. et al.
H3K14ac,H3K18ac,H3K23ac,H3K27me2,H3K36me3,H3K4me1	LeRoy G. et al.
H3K14ac,H3K18ac,H3K23ac,H3K27me2,H3K36me3,H3K4me1,H3K9ac	LeRoy G. et al.
H3K14ac,H3K18ac,H3K23ac,H3K27me2,H3K36me3,H3K4me1,H3K9me1	LeRoy G. et al.
H3K14ac,H3K18ac,H3K23ac,H3K27me2,H3K36me3,H3K4me1,H3K9me2	LeRoy G. et al.
H3K14ac,H3K18ac,H3K23ac,H3K27me2,H3K36me3,H3K4me1,H3K9me3	LeRoy G. et al.
H3K14ac,H3K18ac,H3K23ac,H3K27me2,H3K36me3,H3K4me2,H3K9ac	LeRoy G. et al.
H3K14ac,H3K18ac,H3K23ac,H3K27me2,H3K36me3,H3K4me2,H3K9me1	LeRoy G. et al.
H3K14ac,H3K18ac,H3K23ac,H3K27me2,H3K36me3,H3K4me2,H3K9me2	LeRoy G. et al.
H3K14ac,H3K18ac,H3K23ac,H3K27me2,H3K36me3,H3K4me3,H3K9me2	LeRoy G. et al.
H3K14ac,H3K18ac,H3K23ac,H3K27me2,H3K36me3,H3K9ac	LeRoy G. et al.
H3K14ac,H3K18ac,H3K23ac,H3K27me2,H3K36me3,H3K9me1	LeRoy G. et al.
H3K14ac,H3K18ac,H3K23ac,H3K27me2,H3K36me3,H3K9me2	LeRoy G. et al.
H3K14ac,H3K18ac,H3K23ac,H3K27me2,H3K36me3,H3K9me3	LeRoy G. et al.
H3K14ac,H3K18ac,H3K23ac,H3K27me2,H3K4me1,H3K9me2	Garcia B. et al.
H3K14ac,H3K18ac,H3K23ac,H3K27me2,H3K9me1	LeRoy G. et al.
H3K14ac,H3K18ac,H3K23ac,H3K27me2,H3K9me2	LeRoy G. et al.
H3K14ac,H3K18ac,H3K23ac,H3K27me2,H3K9me3	Garcia B. et al. LeRoy G. et al.
H3K14ac,H3K18ac,H3K23ac,H3K27me3	Garcia B. et al. LeRoy G. et al.
H3K14ac,H3K18ac,H3K23ac,H3K27me3,H3K36me1,H3K4me1	Garcia B. et al. LeRoy G. et al.
H3K14ac,H3K18ac,H3K23ac,H3K27me3,H3K36me1,H3K4me1,H3K9ac	LeRoy G. et al.
H3K14ac,H3K18ac,H3K23ac,H3K27me3,H3K36me1,H3K4me1,H3K9me1	LeRoy G. et al.
H3K14ac,H3K18ac,H3K23ac,H3K27me3,H3K36me1,H3K4me1,H3K9me2	LeRoy G. et al.
H3K14ac,H3K18ac,H3K23ac,H3K27me3,H3K36me1,H3K4me2,H3K9me2	LeRoy G. et al.
H3K14ac,H3K18ac,H3K23ac,H3K27me3,H3K36me1,H3K9ac	LeRoy G. et al.

H3K14ac,H3K18ac,H3K23ac,H3K27me3,H3K36me1,H3K9me1	LeRoy G. et al.
H3K14ac,H3K18ac,H3K23ac,H3K27me3,H3K36me1,H3K9me2	Garcia B. et al. LeRoy G. et al.
H3K14ac,H3K18ac,H3K23ac,H3K27me3,H3K36me1,H3K9me3	Garcia B. et al.
H3K14ac,H3K18ac,H3K23ac,H3K27me3,H3K36me2,H3K4me1,H3K9ac	LeRoy G. et al.
H3K14ac,H3K18ac,H3K23ac,H3K27me3,H3K36me2,H3K4me1,H3K9me1	LeRoy G. et al.
H3K14ac,H3K18ac,H3K23ac,H3K27me3,H3K36me2,H3K4me1,H3K9me2	Garcia B. et al. LeRoy G. et al.
H3K14ac,H3K18ac,H3K23ac,H3K27me3,H3K36me2,H3K4me2,H3K9me1	LeRoy G. et al.
H3K14ac,H3K18ac,H3K23ac,H3K27me3,H3K36me2,H3K4me2,H3K9me2	LeRoy G. et al.
H3K14ac,H3K18ac,H3K23ac,H3K27me3,H3K36me2,H3K4me3,H3K9me1	LeRoy G. et al.
H3K14ac,H3K18ac,H3K23ac,H3K27me3,H3K36me2,H3K9me1	LeRoy G. et al.
H3K14ac,H3K18ac,H3K23ac,H3K27me3,H3K36me2,H3K9me2	LeRoy G. et al.
H3K14ac,H3K18ac,H3K23ac,H3K27me3,H3K36me2,H3K9me3	Garcia B. et al.
H3K14ac,H3K18ac,H3K23ac,H3K27me3,H3K36me3,H3K4me1,H3K9me2	LeRoy G. et al.
H3K14ac,H3K18ac,H3K23ac,H3K27me3,H3K36me3,H3K4me2,H3K9me3	LeRoy G. et al.
H3K14ac,H3K18ac,H3K23ac,H3K27me3,H3K36me3,H3K4me3,H3K9me2	LeRoy G. et al.
H3K14ac,H3K18ac,H3K23ac,H3K27me3,H3K36me3,H3K4me3,H3K9me3	LeRoy G. et al.
H3K14ac,H3K18ac,H3K23ac,H3K27me3,H3K36me3,H3K9me1	LeRoy G. et al.
H3K14ac,H3K18ac,H3K23ac,H3K27me3,H3K4me1	Garcia B. et al.
H3K14ac,H3K18ac,H3K23ac,H3K27me3,H3K4me1,H3K9ac	LeRoy G. et al.
H3K14ac,H3K18ac,H3K23ac,H3K27me3,H3K4me1,H3K9me1	LeRoy G. et al.
H3K14ac,H3K18ac,H3K23ac,H3K27me3,H3K4me1,H3K9me2	Garcia B. et al. LeRoy G. et al.
H3K14ac,H3K18ac,H3K23ac,H3K27me3,H3K9me1	LeRoy G. et al.
H3K14ac,H3K18ac,H3K23ac,H3K27me3,H3K9me2	Garcia B. et al. LeRoy G. et al.
H3K14ac,H3K18ac,H3K23ac,H3K27me3,H3K9me3	LeRoy G. et al.
H3K14ac,H3K18ac,H3K23ac,H3K36me1,H3K9me2	LeRoy G. et al.
H3K14ac,H3K18ac,H3K23ac,H3K36me2,H3K9me1	LeRoy G. et al.
H3K14ac,H3K18ac,H3K23ac,H3K36me2,H3K9me3	Garcia B. et al.
H3K14ac,H3K18ac,H3K23ac,H3K36me3,H3K4me1,H3K9ac	LeRoy G. et al.
H3K14ac,H3K18ac,H3K23ac,H3K36me3,H3K4me1,H3K9me2	LeRoy G. et al.
H3K14ac,H3K18ac,H3K23ac,H3K36me3,H3K4me1,H3K9me3	LeRoy G. et al.
H3K14ac,H3K18ac,H3K23ac,H3K36me3,H3K4me2,H3K9ac	LeRoy G. et al.
H3K14ac,H3K18ac,H3K23ac,H3K36me3,H3K4me3,H3K9me3	LeRoy G. et al.
H3K14ac,H3K18ac,H3K23ac,H3K36me3,H3K9me3	LeRoy G. et al.
H3K14ac,H3K18ac,H3K27me3,H3K36me3,H3K4me1,H3K9me2	LeRoy G. et al.
H3K14ac,H3K23ac	LeRoy G. et al.
H3K14ac,H3K23ac,H3K27me1	LeRoy G. et al.
H3K14ac,H3K23ac,H3K27me1,H3K36me1	Garcia B. et al. LeRoy G. et al.
H3K14ac,H3K23ac,H3K27me1,H3K36me1,H3K9me1	LeRoy G. et al.
H3K14ac,H3K23ac,H3K27me1,H3K36me1,H3K9me3	Garcia B. et al. LeRoy G. et al.
H3K14ac,H3K23ac,H3K27me1,H3K36me2,H3K4me1,H3K9me1	Garcia B. et al.
H3K14ac,H3K23ac,H3K27me1,H3K36me2,H3K4me1,H3K9me2	LeRoy G. et al.
H3K14ac,H3K23ac,H3K27me1,H3K36me2,H3K9me2	Garcia B. et al. LeRoy G. et al.

H3K14ac,H3K23ac,H3K27me1,H3K36me2,H3K9me3	Garcia B. et al. LeRoy G. et al.
H3K14ac,H3K23ac,H3K27me1,H3K36me3,H3K4me1,H3K9me3	LeRoy G. et al.
H3K14ac,H3K23ac,H3K27me1,H3K4me1,H3K9me2	Garcia B. et al.
H3K14ac,H3K23ac,H3K27me1,H3K9me2	Garcia B. et al. LeRoy G. et al.
H3K14ac,H3K23ac,H3K27me1,H3K9me3	LeRoy G. et al.
H3K14ac,H3K23ac,H3K27me2	Garcia B. et al. Garcia B. et al. LeRoy G. et al.
H3K14ac,H3K23ac,H3K27me2,H3K36me1	LeRoy G. et al.
H3K14ac,H3K23ac,H3K27me2,H3K36me1,H3K4me1,H3K9me1	Garcia B. et al.
H3K14ac,H3K23ac,H3K27me2,H3K36me1,H3K9me2	Garcia B. et al. LeRoy G. et al.
H3K14ac,H3K23ac,H3K27me2,H3K36me1,H3K9me3	Garcia B. et al. LeRoy G. et al.
H3K14ac,H3K23ac,H3K27me2,H3K36me2,H3K4me1	Garcia B. et al.
H3K14ac,H3K23ac,H3K27me2,H3K36me2,H3K4me1,H3K9me2	Garcia B. et al. LeRoy G. et al.
H3K14ac,H3K23ac,H3K27me2,H3K36me2,H3K4me1,H3K9me3	LeRoy G. et al.
H3K14ac,H3K23ac,H3K27me2,H3K36me2,H3K9me1	Garcia B. et al.
H3K14ac,H3K23ac,H3K27me2,H3K36me2,H3K9me2	Garcia B. et al. LeRoy G. et al.
H3K14ac,H3K23ac,H3K27me2,H3K36me2,H3K9me3	Garcia B. et al. LeRoy G. et al.
H3K14ac,H3K23ac,H3K27me2,H3K36me3,H3K4me1,H3K9me1	LeRoy G. et al.
H3K14ac,H3K23ac,H3K27me2,H3K36me3,H3K4me1,H3K9me3	LeRoy G. et al.
H3K14ac,H3K23ac,H3K27me2,H3K36me3,H3K9me3	LeRoy G. et al.
H3K14ac,H3K23ac,H3K27me2,H3K4me1,H3K9me2	Garcia B. et al.
H3K14ac,H3K23ac,H3K27me2,H3K9me1	Garcia B. et al. LeRoy G. et al.
H3K14ac,H3K23ac,H3K27me2,H3K9me2	Garcia B. et al. LeRoy G. et al.
H3K14ac,H3K23ac,H3K27me2,H3K9me3	LeRoy G. et al.
H3K14ac,H3K23ac,H3K27me3	Garcia B. et al. LeRoy G. et al.
H3K14ac,H3K23ac,H3K27me3,H3K36me1	LeRoy G. et al.
H3K14ac,H3K23ac,H3K27me3,H3K36me1,H3K4me1,H3K9me1	Garcia B. et al.
H3K14ac,H3K23ac,H3K27me3,H3K36me1,H3K4me1,H3K9me2	LeRoy G. et al.
H3K14ac,H3K23ac,H3K27me3,H3K36me1,H3K9me1	LeRoy G. et al.
H3K14ac,H3K23ac,H3K27me3,H3K36me1,H3K9me2	Garcia B. et al. LeRoy G. et al.
H3K14ac,H3K23ac,H3K27me3,H3K36me1,H3K9me3	Garcia B. et al.
H3K14ac,H3K23ac,H3K27me3,H3K36me2,H3K4me1,H3K9me1	LeRoy G. et al.
H3K14ac,H3K23ac,H3K27me3,H3K36me2,H3K4me1,H3K9me2	Garcia B. et al. LeRoy G. et al.
H3K14ac,H3K23ac,H3K27me3,H3K36me2,H3K9me2	Garcia B. et al. LeRoy G. et al.
H3K14ac,H3K23ac,H3K27me3,H3K36me2,H3K9me3	Garcia B. et al. LeRoy G. et al.

H3K14ac,H3K23ac,H3K27me3,H3K4me1	Garcia B. et al.
H3K14ac,H3K23ac,H3K27me3,H3K4me1,H3K9me1	Garcia B. et al.
H3K14ac,H3K23ac,H3K27me3,H3K4me1,H3K9me2	Garcia B. et al.
H3K14ac,H3K23ac,H3K27me3,H3K9me1	Garcia B. et al. LeRoy G. et al.
H3K14ac,H3K23ac,H3K27me3,H3K9me2	Garcia B. et al. LeRoy G. et al.
H3K14ac,H3K23ac,H3K27me3,H3K9me3	Garcia B. et al. LeRoy G. et al.
H3K14ac,H3K23ac,H3K36me1,H3K9me2	LeRoy G. et al.
H3K14ac,H3K23ac,H3K36me2	LeRoy G. et al.
H3K14ac,H3K23ac,H3K36me2,H3K4me1,H3K9me2	Garcia B. et al.
H3K14ac,H3K23ac,H3K36me2,H3K9me2	Garcia B. et al.
H3K14ac,H3K23ac,H3K36me2,H3K9me3	LeRoy G. et al.
H3K14ac,H3K23ac,H3K36me3,H3K9me3	LeRoy G. et al.
H3K14ac,H3K23ac,H3K4me1,H3K9me2	Garcia B. et al.
H3K14ac,H3K23ac,H3K9me2	Garcia B. et al.
H3K14ac,H3K23ac,H3K9me3	Garcia B. et al.
H3K14ac,H3K27me1,H3K36me1,H3K4me1,H3K9me2	Garcia B. et al.
H3K14ac,H3K27me1,H3K36me1,H3K9me3	LeRoy G. et al.
H3K14ac,H3K27me1,H3K36me2,H3K4me1,H3K9me2	Garcia B. et al.
H3K14ac,H3K27me1,H3K36me2,H3K9me2	Garcia B. et al.
H3K14ac,H3K27me1,H3K36me2,H3K9me3	Garcia B. et al. LeRoy G. et al.
H3K14ac,H3K27me1,H3K9me1	Garcia B. et al.
H3K14ac,H3K27me1,H3K9me2	Garcia B. et al.
H3K14ac,H3K27me1,H3K9me3	Garcia B. et al. LeRoy G. et al.
H3K14ac,H3K27me2	Garcia B. et al.
H3K14ac,H3K27me2,H3K36me1,H3K9me2	Garcia B. et al. LeRoy G. et al.
H3K14ac,H3K27me2,H3K36me1,H3K9me3	LeRoy G. et al.
H3K14ac,H3K27me2,H3K36me2	Garcia B. et al.
H3K14ac,H3K27me2,H3K36me2,H3K4me1,H3K9me2	Garcia B. et al. LeRoy G. et al.
H3K14ac,H3K27me2,H3K36me2,H3K9me2	Garcia B. et al. LeRoy G. et al.
H3K14ac,H3K27me2,H3K36me2,H3K9me3	Garcia B. et al. LeRoy G. et al.
H3K14ac,H3K27me2,H3K36me3,H3K4me1,H3K9me1	LeRoy G. et al.
H3K14ac,H3K27me2,H3K36me3,H3K4me1,H3K9me2	LeRoy G. et al.
H3K14ac,H3K27me2,H3K36me3,H3K9me2	LeRoy G. et al.
H3K14ac,H3K27me2,H3K9me1	Garcia B. et al.
H3K14ac,H3K27me2,H3K9me2	Garcia B. et al. LeRoy G. et al.
H3K14ac,H3K27me2,H3K9me3	Garcia B. et al. LeRoy G. et al.
H3K14ac,H3K27me3,H3K36me1	Garcia B. et al.
H3K14ac,H3K27me3,H3K36me1,H3K9me2	LeRoy G. et al.

H3K14ac,H3K27me3,H3K36me2,H3K4me1,H3K9me1	LeRoy G. et al.
H3K14ac,H3K27me3,H3K36me2,H3K4me1,H3K9me2	Garcia B. et al. LeRoy G. et al.
H3K14ac,H3K27me3,H3K36me2,H3K9me2	Garcia B. et al. LeRoy G. et al.
H3K14ac,H3K27me3,H3K36me2,H3K9me3	Garcia B. et al. LeRoy G. et al.
H3K14ac,H3K27me3,H3K4me1	Garcia B. et al.
H3K14ac,H3K27me3,H3K4me1,H3K9me1	Garcia B. et al.
H3K14ac,H3K27me3,H3K9me2	Garcia B. et al. LeRoy G. et al.
H3K14ac,H3K27me3,H3K9me3	LeRoy G. et al.
H3K14ac,H3K36me1,H3K4me1,H3K9me2	Garcia B. et al.
H3K14ac,H3K36me1,H3K9me3	Garcia B. et al.
H3K14ac,H3K36me2	Garcia B. et al.
H3K14ac,H3K36me2,H3K9me2	Garcia B. et al.
H3K14ac,H3K36me2,H3K9me3	LeRoy G. et al.
H3K14ac,H3K4me1,H3K9me2	Garcia B. et al.
H3K14ac,H3K9me2	Garcia B. et al.
H3K18ac,H3K23ac,H3K27me3,H3K4me1,H3K9me2	Garcia B. et al.
H3K18ac,H3K27me2,H3K9me2	Garcia B. et al.
H3K23ac	LeRoy G. et al.
H3K23ac,H3K27me1	Garcia B. et al.
H3K23ac,H3K27me1,H3K36me1,H3K9me3	LeRoy G. et al.
H3K23ac,H3K27me1,H3K36me3,H3K9me3	LeRoy G. et al.
H3K23ac,H3K27me1,H3K9me2	Garcia B. et al. LeRoy G. et al.
H3K23ac,H3K27me1,H3K9me3	LeRoy G. et al.
H3K23ac,H3K27me2	Garcia B. et al. LeRoy G. et al.
H3K23ac,H3K27me2,H3K36me1	LeRoy G. et al.
H3K23ac,H3K27me2,H3K36me1,H3K9me2	Garcia B. et al.
H3K23ac,H3K27me2,H3K36me1,H3K9me3	Garcia B. et al.
H3K23ac,H3K27me2,H3K36me2,H3K9me2	Garcia B. et al.
H3K23ac,H3K27me2,H3K36me2,H3K9me3	Garcia B. et al. LeRoy G. et al.
H3K23ac,H3K27me2,H3K4me1	Garcia B. et al.
H3K23ac,H3K27me2,H3K9me1	Garcia B. et al. LeRoy G. et al.
H3K23ac,H3K27me2,H3K9me2	Garcia B. et al. LeRoy G. et al.
H3K23ac,H3K27me2,H3K9me3	Garcia B. et al. LeRoy G. et al.
H3K23ac,H3K27me3	Garcia B. et al. LeRoy G. et al.
H3K23ac,H3K27me3,H3K36me1,H3K9me2	Garcia B. et al.
H3K23ac,H3K27me3,H3K36me2,H3K9me2	Garcia B. et al.
H3K23ac,H3K27me3,H3K9me1	Garcia B. et al. LeRoy G. et al.

H3K23ac,H3K27me3,H3K9me2	Garcia B. et al. LeRoy G. et al.
H3K23ac,H3K27me3,H3K9me3	Garcia B. et al.
H3K23ac,H3K36me2,H3K9me3	Garcia B. et al. LeRoy G. et al.
H3K23ac,H3K4me1,H3K9me2	Garcia B. et al.
H3K23ac,H3K9me2	Garcia B. et al.
H3K23ac,H3K9me3	Garcia B. et al.
H3K27me1	Garcia B. et al. LeRoy G. et al.
H3K27me1,H3K36me1,H3K9me2	Garcia B. et al.
H3K27me1,H3K36me1,H3K9me3	LeRoy G. et al.
H3K27me1,H3K36me2	Garcia B. et al.
H3K27me1,H3K36me2,H3K9me1	Garcia B. et al.
H3K27me1,H3K36me2,H3K9me2	Garcia B. et al.
H3K27me1,H3K36me2,H3K9me3	Garcia B. et al. LeRoy G. et al.
H3K27me1,H3K9me1	Garcia B. et al.
H3K27me1,H3K9me2	Garcia B. et al. LeRoy G. et al.
H3K27me1,H3K9me3	Garcia B. et al. LeRoy G. et al.
H3K27me2	Garcia B. et al.
H3K27me2,H3K36me1,H3K9me2	LeRoy G. et al.
H3K27me2,H3K36me1,H3K9me3	LeRoy G. et al.
H3K27me2,H3K36me2	Garcia B. et al.
H3K27me2,H3K36me2,H3K4me1,H3K9me2	LeRoy G. et al.
H3K27me2,H3K36me2,H3K9me2	Garcia B. et al. LeRoy G. et al.
H3K27me2,H3K36me2,H3K9me3	Garcia B. et al. LeRoy G. et al.
H3K27me2,H3K36me3,H3K4me1,H3K9me1	LeRoy G. et al.
H3K27me2,H3K36me3,H3K4me1,H3K9me2	LeRoy G. et al.
H3K27me2,H3K9me1	Garcia B. et al.
H3K27me2,H3K9me2	Garcia B. et al. LeRoy G. et al.
H3K27me2,H3K9me3	Garcia B. et al. LeRoy G. et al.
H3K27me3	Garcia B. et al.
H3K27me3,H3K36me1,H3K4me1,H3K9me2	LeRoy G. et al.
H3K27me3,H3K36me1,H3K9me2	Garcia B. et al. LeRoy G. et al.
H3K27me3,H3K36me1,H3K9me3	LeRoy G. et al.
H3K27me3,H3K36me2,H3K4me1,H3K9me1	LeRoy G. et al.
H3K27me3,H3K36me2,H3K9me1	Garcia B. et al.
H3K27me3,H3K36me2,H3K9me2	Garcia B. et al. LeRoy G. et al.
H3K27me3,H3K36me2,H3K9me3	LeRoy G. et al.
H3K27me3,H3K9me1	Garcia B. et al.

H3K27me3,H3K9me2	Garcia B. et al. LeRoy G. et al.
H3K27me3,H3K9me3	Garcia B. et al. LeRoy G. et al.
H3K36me1	Garcia B. et al.
H3K36me1,H3K9me2	LeRoy G. et al.
H3K36me2	Garcia B. et al.
H3K36me2,H3K9me1	Garcia B. et al.
H3K36me2,H3K9me2	Garcia B. et al.
H3K36me2,H3K9me3	Garcia B. et al.
H3K9me1	Garcia B. et al.
H3K9me2	Garcia B. et al. LeRoy G. et al.
H3K9me3	Garcia B. et al.
H4K12ac	Phanstiel D. et al. Pesavento J. et al.
H4K12ac,H4K16ac	Phanstiel D. et al. Pesavento J. et al.
H4K12ac,H4K16ac,H4K20me1	Phanstiel D. et al. Pesavento J. et al.
H4K12ac,H4K16ac,H4K20me1,H4K5ac	Phanstiel D. et al. Pesavento J. et al.
H4K12ac,H4K16ac,H4K20me1,H4K5ac,H4K8ac	Phanstiel D. et al. Pesavento J. et al.
H4K12ac,H4K16ac,H4K20me1,H4K8ac	Phanstiel D. et al. Pesavento J. et al.
H4K12ac,H4K16ac,H4K20me2	Phanstiel D. et al. Pesavento J. et al.
H4K12ac,H4K16ac,H4K20me2,H4K5ac	Phanstiel D. et al. Pesavento J. et al.
H4K12ac,H4K16ac,H4K20me2,H4K5ac,H4K8ac	Phanstiel D. et al. Pesavento J. et al.
H4K12ac,H4K16ac,H4K20me2,H4K5ac,H4K8ac,H4R3me1	Phanstiel D. et al.
H4K12ac,H4K16ac,H4K20me2,H4K5ac,H4R3me1	Phanstiel D. et al.
H4K12ac,H4K16ac,H4K20me2,H4K8ac	Phanstiel D. et al. Pesavento J. et al.
H4K12ac,H4K16ac,H4K20me2,H4K8ac,H4R3me1	Phanstiel D. et al.
H4K12ac,H4K16ac,H4K20me2,H4R3me1	Phanstiel D. et al. Pesavento J. et al.
H4K12ac,H4K16ac,H4K20me2,H4R3me2	Pesavento J. et al.
H4K12ac,H4K16ac,H4K20me3	Phanstiel D. et al.
H4K12ac,H4K16ac,H4K20me3,H4K5ac	Phanstiel D. et al.
H4K12ac,H4K16ac,H4K20me3,H4K5ac,H4K8ac	Phanstiel D. et al.
H4K12ac,H4K16ac,H4K20me3,H4K8ac	Phanstiel D. et al.
H4K12ac,H4K16ac,H4K5ac	Phanstiel D. et al. Pesavento J. et al.
H4K12ac,H4K16ac,H4K5ac,H4K8ac	Phanstiel D. et al. Pesavento J. et al.
H4K12ac,H4K16ac,H4K8ac	Phanstiel D. et al.

	Pesavento J. et al.
H4K12ac,H4K20me1	Pesavento J. et al.
H4K12ac,H4K20me1,H4K5ac	Pesavento J. et al.
H4K12ac,H4K20me1,H4K5ac,H4K8ac	Phanstiel D. et al.
H4K12ac,H4K20me1,H4K8ac	Pesavento J. et al.
H4K12ac,H4K20me2	Phanstiel D. et al.<br>Pesavento J. et al.
H4K12ac,H4K20me2,H4K5ac	Pesavento J. et al.
H4K12ac,H4K20me2,H4K5ac,H4K8ac	Phanstiel D. et al.
H4K12ac,H4K20me2,H4K5ac,H4K8ac,H4R3me1	Phanstiel D. et al.
H4K12ac,H4K20me2,H4K8ac	Phanstiel D. et al.<br>Pesavento J. et al.
H4K12ac,H4K20me2,H4R3me1	Phanstiel D. et al.
H4K12ac,H4K20me3	Phanstiel D. et al.
H4K12ac,H4K20me3,H4K5ac,H4K8ac	Phanstiel D. et al.
H4K12ac,H4K5ac	Pesavento J. et al.
H4K12ac,H4K5ac,H4K8ac	Phanstiel D. et al.
H4K12ac,H4K8ac	Pesavento J. et al.
H4K16ac	Phanstiel D. et al.<br>Pesavento J. et al.
H4K16ac,H4K20me1	Phanstiel D. et al.<br>Pesavento J. et al.
H4K16ac,H4K20me1,H4K5ac	Phanstiel D. et al.<br>Pesavento J. et al.
H4K16ac,H4K20me1,H4K5ac,H4K8ac	Phanstiel D. et al.
H4K16ac,H4K20me1,H4K8ac	Phanstiel D. et al.<br>Pesavento J. et al.
H4K16ac,H4K20me2	Phanstiel D. et al.<br>Pesavento J. et al.
H4K16ac,H4K20me2,H4K5ac	Phanstiel D. et al.<br>Pesavento J. et al.
H4K16ac,H4K20me2,H4K5ac,H4K8ac	Phanstiel D. et al.
H4K16ac,H4K20me2,H4K5ac,H4K8ac,H4R3me1	Phanstiel D. et al.
H4K16ac,H4K20me2,H4K5ac,H4R3me1	Pesavento J. et al.
H4K16ac,H4K20me2,H4K5ac,H4R3me2	Pesavento J. et al.
H4K16ac,H4K20me2,H4K8ac	Phanstiel D. et al.<br>Pesavento J. et al.
H4K16ac,H4K20me2,H4K8ac,H4R3me1	Pesavento J. et al.
H4K16ac,H4K20me2,H4K8ac,H4R3me2	Pesavento J. et al.
H4K16ac,H4K20me2,H4R3me1	Phanstiel D. et al.<br>Pesavento J. et al.
H4K16ac,H4K20me2,H4R3me2	Phanstiel D. et al.<br>Pesavento J. et al.
H4K16ac,H4K20me2,H4S1P	Phanstiel D. et al.
H4K16ac,H4K20me3	Phanstiel D. et al.<br>Pesavento J. et al.
H4K16ac,H4K20me3,H4K5ac,H4K8ac	Phanstiel D. et al.
H4K16ac,H4K20me3,H4S1P	Phanstiel D. et al.
H4K16ac,H4K5ac	Phanstiel D. et al.

	Pesavento J. et al.
H4K16ac,H4K5ac,H4K8ac	Phanstiel D. et al.
H4K16ac,H4K8ac	Phanstiel D. et al.<br>Pesavento J. et al.
H4K20me1	Phanstiel D. et al.<br>Pesavento J. et al.
H4K20me1,H4K5ac	Phanstiel D. et al.<br>Pesavento J. et al.
H4K20me1,H4K5ac,H4K8ac	Phanstiel D. et al.
H4K20me1,H4K8ac	Pesavento J. et al.
H4K20me2	Phanstiel D. et al.<br>Pesavento J. et al.
H4K20me2,H4K5ac	Phanstiel D. et al.<br>Pesavento J. et al.
H4K20me2,H4K5ac,H4K8ac	Phanstiel D. et al.
H4K20me2,H4K5ac,H4K8ac,H4R3me1	Phanstiel D. et al.
H4K20me2,H4K5ac,H4R3me1	Phanstiel D. et al.
H4K20me2,H4K8ac	Phanstiel D. et al.<br>Pesavento J. et al.
H4K20me2,H4K8ac,H4R3me1	Phanstiel D. et al.
H4K20me2,H4R3me1	Phanstiel D. et al.<br>Pesavento J. et al.
H4K20me2,H4R3me2	Pesavento J. et al.
H4K20me2,H4S1P	Phanstiel D. et al.
H4K20me3	Phanstiel D. et al.<br>Pesavento J. et al.
H4K20me3,H4K5ac	Phanstiel D. et al.
H4K20me3,H4K5ac,H4K8ac	Phanstiel D. et al.
H4K20me3,H4K8ac	Phanstiel D. et al.
H4K20me3,H4S1P	Phanstiel D. et al.
H4K5ac	Phanstiel D. et al.<br>Pesavento J. et al.
H4K5ac,H4K8ac	Phanstiel D. et al.
H4K8ac	Phanstiel D. et al.<br>Pesavento J. et al.
H4S1P	Phanstiel D. et al.

Table S2. Number and fraction of distal p300 peaks that have only one histone modification peak.

	B Cell (%)	T Cell (%)	ES Cell (%)
**H3K4me1**	193 (4.5)	78 (3.7)	222 (8.1)
**H3K4me2**	131 (3.0)	8 (0.4)	33 (1.2)
**H3K4me3**	16 (0.4)	83 (4.0)	2 (0.1)
**H3K9ac**	72 (1.7)	5 (0.2)	2 (0.1)
**H3K27ac**	143 (3.3)	36 (1.7)	65 (2.4)

Table S3. Overlap between B cell enhancers from the biclusters in this study and those reported in Ernst et al. [5]. P-values are calculated using cumulative hypergeometric distribution. We use a set of codes to represent these states in a compact and simply way. For instance, the code M12A9 represents the histone mark combination of H3K4me1, H3K4me2, and H3K9ac. The same sets of codes apply to all supplemental tables.

Bicluster ID	# enhancers in bicluster	# overlapped enhancer	p-value
M12	351	250	0.71
M13	51	30	0.99
M1A9	152	133	4.06E-6
M1A27	505	470	3.08E-35
M23	636	416	1
M2A9	1172	945	1.85E-14
M2A27	1442	1189	2.70E-27
M3A9	394	241	1
M3A27	471	323	0.98
A9A27	1270	1016	8.40E-14
M123	18	11	0.91
M12A9	54	47	0.01
M12A27	119	109	1.25E-7
M13A9	11	6	0.95
M13A27	12	10	0.31
M1A927	81	77	1.37E-7
M23A9	219	127	1
M23A27	251	166	0.99
M2A927	629	500	5.60E-6
M3A927	207	120	1
M12A927	33	31	0.002
M23A927	141	81	1

Table S4. Overlap between ES cell enhancers from the biclusters in this study and those reported in Ernst et al. [5]. P-values are calculated using cumulative hypergeometric distribution.

Bicluster ID	# enhancers in bicluster	# overlapped enhancer	p-value
M12	447	129	5.05E-4
M13	33	13	0.02
M1A9	127	61	1.04E-10
M1A27	664	221	2.23E-13
M23	371	137	1.28E-11
M2A9	342	137	6.61E-15
M2A27	1136	338	9.11E-14
M3A9	153	55	8.55E-5
M3A27	193	51	0.12
M123	22	8	0.10
M12A9	46	26	5.65E-7
M12A27	222	86	1.29E-8
M13A9	12	5	0.11
M13A27	17	7	0.07
M1A927	47	28	4.26E-8
M23A9	109	33	0.04
M23A27	144	44	0.015
M2A927	140	64	4.94E-10
M3A927	69	24	0.01
M123A27	14	5	0.19
M12A927	26	16	2.14E-5
M23A927	56	18	0.06

Table S5. Overlap between enhancer biclusters that have more H3K4me1 mark and Premod cis-regulatory modules. Shown are hypergeometric p-values for the overlaps in all three cell types. Most H3K4me1-negative biclusters in ES cells do not significantly overlap with PreMod CRMs. We believe this is due to the limitation of PreMod because DNA motifs of many ESC-specific TFs were not used in the generation of PreMod CRMs. "-" indicates no bicluster was found.

Bicluster ID	B Cell	T Cell	ES Cell
M23	0.00278	0	0.2815
M2A9	0.000434	0	0.9986
M2A27	0.347443	0	1
M3A9	0.013469	0	0.5096
M3A27	0.001594	0	0.2284
A927	0.170946	0	-
M23A9	0.000298	0.0017	0.045
M23A27	0.03447	0.0001	0.1536
M2A927	0.008988	0	0.8671
M3A927	0.005003	0	0.3515
M23A927	0.038118	0.033	0.2586

References

1. Garcia BA, Pesavento JJ, Mizzen CA, Kelleher NL: Pervasive combinatorial modification of histone H3 in human cells. *Nat Methods* 2007, 4(6):487-489.

2. LeRoy G, Weston JT, Zee BM, Young NL, Plazas-Mayorca MD, Garcia BA: Heterochromatin protein 1 is extensively decorated with histone code-like post-translational modifications. *Mol Cell Proteomics* 2009, 8(11):2432-2442.

3. Phanstiel D, Brumbaugh J, Berggren WT, Conard K, Feng X, Levenstein ME, McAlister GC, Thomson JA, Coon JJ: Mass spectrometry identifies and quantifies 74 unique histone H4 isoforms in differentiating human embryonic stem cells. *Proc Natl Acad Sci U S A* 2008, 105(11):4093-4098.

4. Pesavento JJ, Bullock CR, LeDuc RD, Mizzen CA, Kelleher NL: Combinatorial modification of human histone H4 quantitated by two-dimensional liquid chromatography coupled with top down mass spectrometry. *J Biol Chem* 2008, 283(22):14927-14937.

5. Ernst J, Kheradpour P, Mikkelsen TS, Shoresh N, Ward LD, Epstein CB, Zhang X, Wang L, Issner R, Coyne M *et al*: Mapping and analysis of chromatin state dynamics in nine human cell types. *Nature* 2011, 473(7345):43-49.
