# Supplementary material for: Finding combinatorial histone code by semi-supervised biclustering
Source: BMC Genomics. 2012 Jul 3;13:301. doi: 10.1186/1471-2164-13-301 (PMC3443427; doi:10.1186/1471-2164-13-301)

Figure S1. Venn diagram of enhancers in three cell types used as inputs to the biclustering algorithms. Enhancers were identified as distal p300 ChIP-Seq peaks.

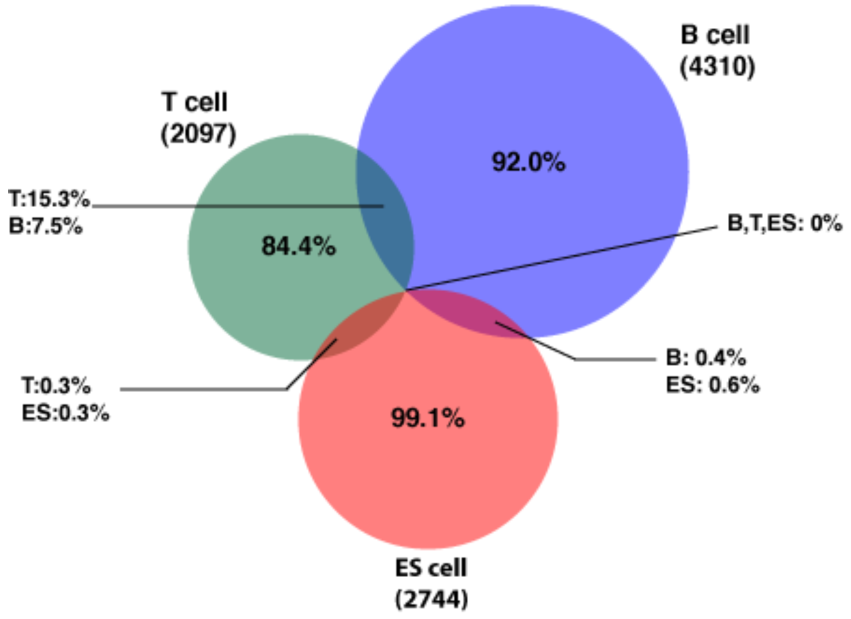

Figure S3. Promoter coverage of all 26 possible histone states involving at least two histone modification marks. Y-axis, percentage of enhancers in biclusters that are associated with a given histone state. Histone marks of each state is indicated at the bottom of the histogram. Each column represents a histone state with the filled square indicating the presence of a histone mark in the state and empty square otherwise.

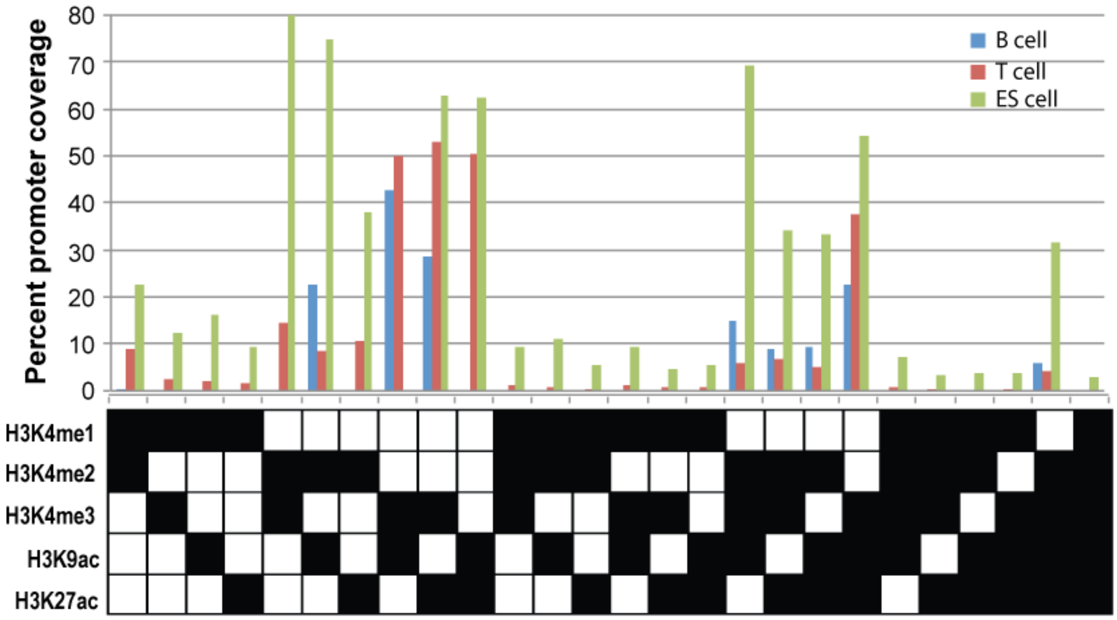

Supplement: Additional file 2 — Figure S1. Venn diagram of enhancers in three cell types used as inputs to the biclustering algorithms. Figure S3. Promoter coverage of all 26 possible histone states involving at least two histone modification marks. [file 1471-2164-13-301-S2.pdf]
