# Supplementary material for: Finding combinatorial histone code by semi-supervised biclustering
Source: BMC Genomics. 2012 Jul 3;13:301. doi: 10.1186/1471-2164-13-301 (PMC3443427; doi:10.1186/1471-2164-13-301)
Supplement: Additional file 3 — Table S6. Histone states of enhancers as defined by biclustering in B, T, and ES cells. [file 1471-2164-13-301-S3.pdf]

Figure S2. Pseudo code for the semi-supervised CoSBI algorithm

Three dimensions of the input GCP matrix are Genomic loci, Chromatin modifications, and ChIP-seq signal Positions. Output is the set of biclusters.

Input: ***3D GCP*** *data, prior co-occurrence probability matrix* ***P***

$\alpha,\ \beta,\ min_g,\ min_s$

Output: *set of coherernt biclusters* $\{Bi_i\}$

Begin

for each genomic locus{

generate histone mark similarity C;

$D_{ij} = 1 - C_{ij}$ *; //convert similarity matrix to distance matrix*

$D'_{ij} = D_{ij} \times (1 - P_{ij})$ *; //incorporate prior knowledge to the biclustering process*

D' = *all-pair-shortest-path*(D') *; // update the distance matrix*

$C'_{ij} = 1 - D'_{ij}$ *; // convert distance matrix back to similarity matrix*

maximal_sample_sets=depth-first-search(C', β) *; //identify all maximal sample sets*

}

$\{Bi_i\}$*=tree_enumeration({maximal_sample_sets},* α, min_g, min_s*)*

$\{Bi_i\}$*=merging*$\{Bi_i\}$*;*

End
